# Serum Uromodulin and All-Cause Mortality in Peritoneal Dialysis Patients: A Chinese Cohort Study

**DOI:** 10.1016/j.xkme.2022.100536

**Published:** 2022-08-23

**Authors:** Dominik Steubl, Li Fan, Yunfang Zhang, Fei Xiong, Hongbo Li, Hao Zhang, Jing Hu, Amy B. Karger, Lesley A. Inker, Xueqing Yu, Andrew S. Levey

**Affiliations:** 1 Department of Nephrology, Klinikum rechts der Isar, Technical University Munich, Munich, Germany; 2 Division of Nephrology, Tufts Medical Center, Boston, Massachusetts; 3 Department of Nephrology, The First Affiliated Hospital of Sun Yat-sen University, NHC Key Laboratory of Nephrology (Sun Yat-sen University), Guangdong Provincial Key Laboratory of Nephrology, Guangzhou, China; 4 Department of Nephrology, Huadu District People’s Hospital of Guangzhou, Huadu, China; 5 Department of Nephrology, Wuhan No.1 Hospital and Wuhan Hospital of Traditional Chinese and Western Medicine, Wuhan, China; 6 Department of Nephrology, The Third Xiangya Hospital of Central South University, Changsha, China; 7 Department of Laboratory Medicine and Pathology, University of Minnesota, Minneapolis, Minnesota; 8 Department of Nephrology, Guangdong Provincial People’s Hospital and Guangdong Academy of Medical Sciences, Guangzhou, China

To the Editor:

Uromodulin is exclusively expressed and secreted by cells of the ascending limb of the loop of Henle and the distal tubule into the urine and the bloodstream and is hypothesized to be a marker for overall nephron/tubular mass.^1^^,^^2^ Serum uromodulin (sUMOD) concentration correlates positively with estimated glomerular filtration rate, and lower sUMOD is associated with higher risk of mortality in various populations.^3^, ^4^, ^5^ The range of sUMOD concentrations, its correlation with residual kidney function, and its association with mortality in patients with kidney failure have not been studied. We hypothesized that lower sUMOD concentration would be associated with lower residual kidney function and higher mortality in patients receiving peritoneal dialysis.

In this work, we evaluated these associations in a subgroup of 936 individuals from a previously described cohort of patients with kidney failure treated with continuous ambulatory peritoneal dialysis (CAPD) from Guangzhou, China.^6^ We selected all patients for whom a serum sample at baseline was available. All patients provided written informed consent before participation. Local institutional review boards approved the study methods (approval NO: [2018]22). The study adheres to the Declaration of Helsinki.

sUMOD measurements were performed as previously described.^4^ The primary outcome was all-cause mortality, obtained by review of medical records and telephone interviews. A brief description of the statistical analyses can be found in Item S1.

Mean age of the cohort was 50 ± 15 years, and 48% were female (Table S1). Patients had been treated with CAPD for a median [25^th^, 75^th^ percentiles] of 15.8 [2.2, 35.5] months before study enrollment. Median residual urine volume was 500 [150, 900] mL/day and residual kidney function was 1.6 [0.3, 3.6] mL/min/1.73 m^2^. The median sUMOD level was 9.2 [4.5, 15.2] ng/mL (Fig 1). There were no significant differences in baseline variables across sUMOD quartiles (Table S1). Furthermore, sUMOD did not correlate significantly with residual kidney function (correlation coefficient *r* = -0.03, *P* = 0.31, Fig S1).

**Figure 1:**
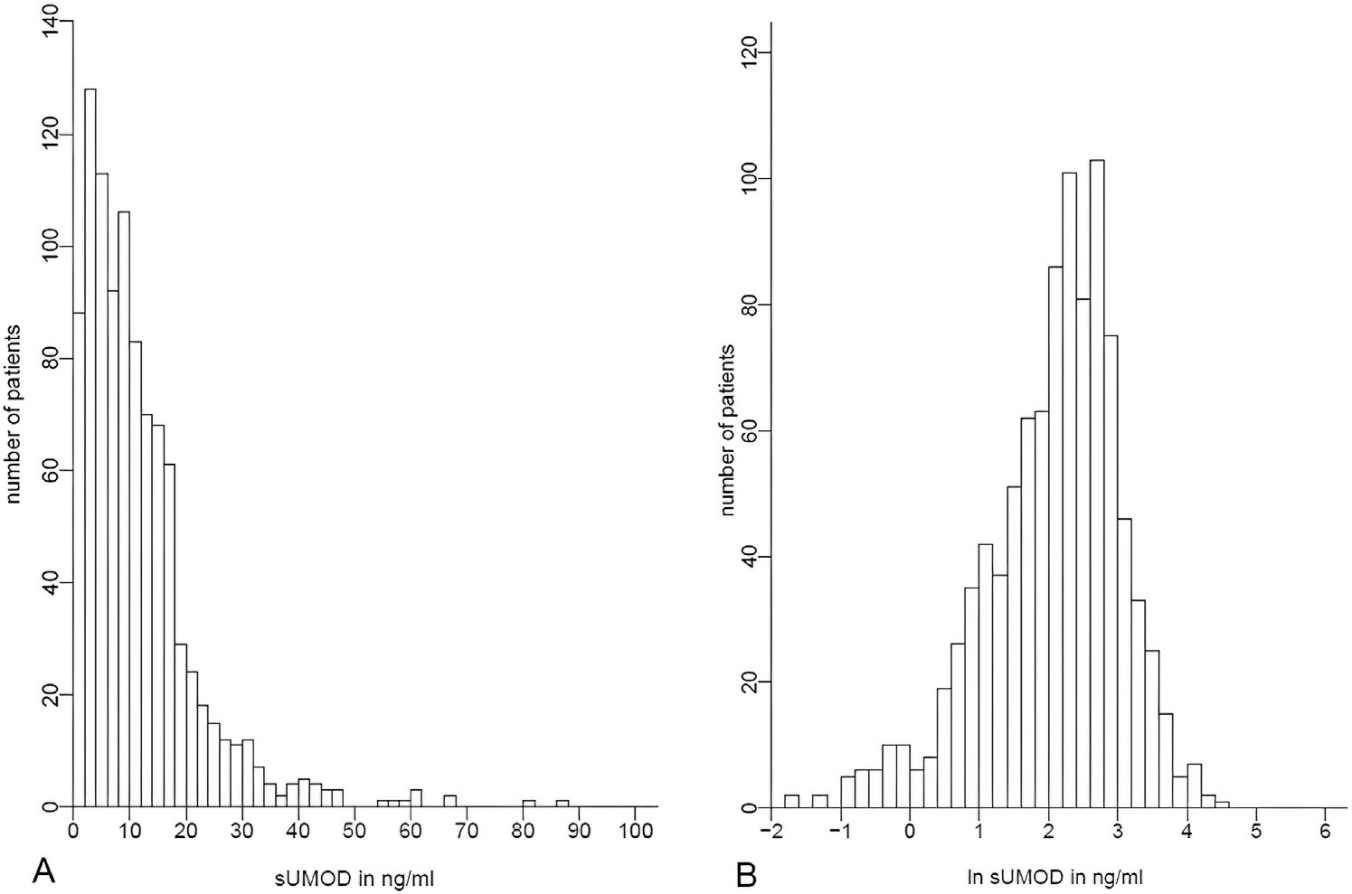
Histograms showing the distribution of serum uromodulin concentration on a (A) raw scale and (B) natural logarithmic scale. One patient with a sUMOD concentration >100 ng/mL was excluded from this figure for illustrative purposes. sUMOD, serum uromodulin.

A total of 195 (20.8%) participants died during a median follow-up interval of 46.8 [25.9, 54.0] months, and the number and rate of events was comparable across all sUMOD quartiles (Table S2 and Fig S2). Mortality rates were higher versus sUMOD quartiles in this study than in the German chronic kidney disease cohort study^4^ (Table S2). In multivariable Cox regression analysis, sUMOD was not associated with all-cause mortality (hazard ratio, 0.97; 95% confidence interval, 0.84-1.13 per one unit higher log sUMOD and 1.03 [0.69-1.55] for uromodulin Q4 vs Q1) (Table 1). Consistent results were observed with cardiovascular and noncardiovascular mortality (Table S3).

**Table 1:** Cox Regression Analysis to Evaluate the Association of Serum Uromodulin With All-Cause Mortality in Patients Treated with Peritoneal Dialysis (n=936)

	Events (%)	Univariable	Model 1^a^	Model 2^b^	Model 3^c^
Effect per each one unit increase in log serum uromodulin	195 (20.8%)	1.03 (0.90-1.19)	0.98 (0.84-1.14)	0.97 (0.83-1.12)	0.97 (0.84-1.13)
Q1	47 (20.7%)	Reference	Reference	Reference	Reference
Q2	51 (21.3%)	1.03 (0.69-1.53)	0.94 (0.63-1.40)	0.93 (0.62-1.40)	0.94 (0.63-1.41)
Q3	47 (20.0%)	0.92 (0.61-1.38)	0.74 (0.49-1.22)	0.74 (0.49-1.12)	0.74 (0.49-1.13)
Q4	50 (21.4%)	1.07 (0.72-1.60)	1.06 (0.71-1.58)	1.02 (0.68-1.54)	1.03 (0.69-1.55)

Results are presented as hazard ratios with 95% confidence intervals given in parentheses. Serum uromodulin was evaluated on a logarithmic scale as a continuous variable and on a raw scale for categorization into quartiles.

There was no correlation between serum uromodulin level and duration of peritoneal dialysis (correlation coefficient *r* = 0.04; 95% CI, -0.02 to 0.11; *P* = 0.19).

Serum uromodulin quartile distribution: Quartile 1 (Q1) < 4.5 ng/mL, Quartile 2 (Q2) ≥ 4.5 and < 9.2 ng/mL, Quartile 3 (Q3) ≥ 9.2 and < 15.225 ng/mL, Quartile 4 (Q4) ≥ 15.225 ng/mL.

a adjusted for age, sex, body mass index, diabetes, systolic blood pressure, serum phosphorus, serum potassium, serum albumin, serum C-reactive protein, serum total cholesterol

b Model 1 + peritoneal ultrafiltration, peritoneal average mean of urea and creatinine clearance, renal average mean of urea and creatinine clearance

c Model 2 + dialysis vintage

Our findings are in contrast to previous studies demonstrating both a strong correlation of sUMOD with estimated glomerular filtration rate, ie, higher in healthy individuals versus those with chronic kidney disease, as well as an association with mortality.^3^^,^^4^ There are various potential explanations: first, analytical limitations might have led to inaccurate measurement of sUMOD at very low concentrations close to the lower limit of detection (2.0 ng/mL) of the assay, and other uremic toxins might have interfered with the assay. Second, little is known about a potential intraday or day-to-day variability of uromodulin secretion into the blood, so assessment of sUMOD levels by one measurement might not reliably reflect average levels. Also, sUMOD concentrations in our study were much lower compared with previous studies and absolute differences among patients were smaller,^3^^,^^4^ therefore, we might not have been able to detect a contribution of sUMOD to mortality risk in the patients included. Last, because the assay is not validated for peritoneal dialysate and we therefore did not measure sUMOD concentrations in the peritoneal dialysis fluid, we cannot rule out a potential influence of peritoneal dialysis treatment on serum sUMOD concentrations.

To our knowledge this is the first cohort with a large number of patients receiving CAPD and sufficient follow-up time in which the association of sUMOD with residual kidney function and mortality has been assessed. This allowed us to perform a thorough multivariable analysis, adjusting for a large set of covariables that is associated with mortality in patients receiving CAPD. However, we only included patients treated with CAPD, so no inferences can be made regarding patients treated with automated peritoneal dialysis or hemodialysis. We only measured uromodulin in a single specimen, so the impact of natural variability of sUMOD concentrations could have impacted the results at these low levels. The cohort consisted of Chinese participants only, which limits the comparability and generalizability of sUMOD concentrations and outcomes to other populations and published data, eg, in patients with chronic kidney disease, who are predominantly White.

In conclusion, sUMOD levels were substantially lower in kidney failure patients receiving CAPD compared with patients with chronic kidney disease, likely reflecting low nephron/tubular mass. In addition, sUMOD was not associated with residual kidney function and mortality in these patients and can therefore not be recommended as a prognostic biomarker for mortality in this population.

## Supplementary Material

Supplementary File (PDF) Figs S1-S2; Item S1; Tables S1-S3.
