# Supplementary material for: Serum Uromodulin and All-Cause Mortality in Peritoneal Dialysis Patients: A Chinese Cohort Study
Source: Kidney Med. 2022 Aug 23;4(10):100536. doi: 10.1016/j.xkme.2022.100536 (PMC9577049; doi:10.1016/j.xkme.2022.100536)
Supplement: Supplementary File (PDF) — Figs S1-S2; Item S1; Tables S1-S3. [file mmc1.pdf]

Figure S1: Correlation between serum uromodulin concentrations and residual kidney function

Line was the least squares line and the beta value was the β of RKF from a linear model. Now we present the least squares line to be consistent with the beta value.

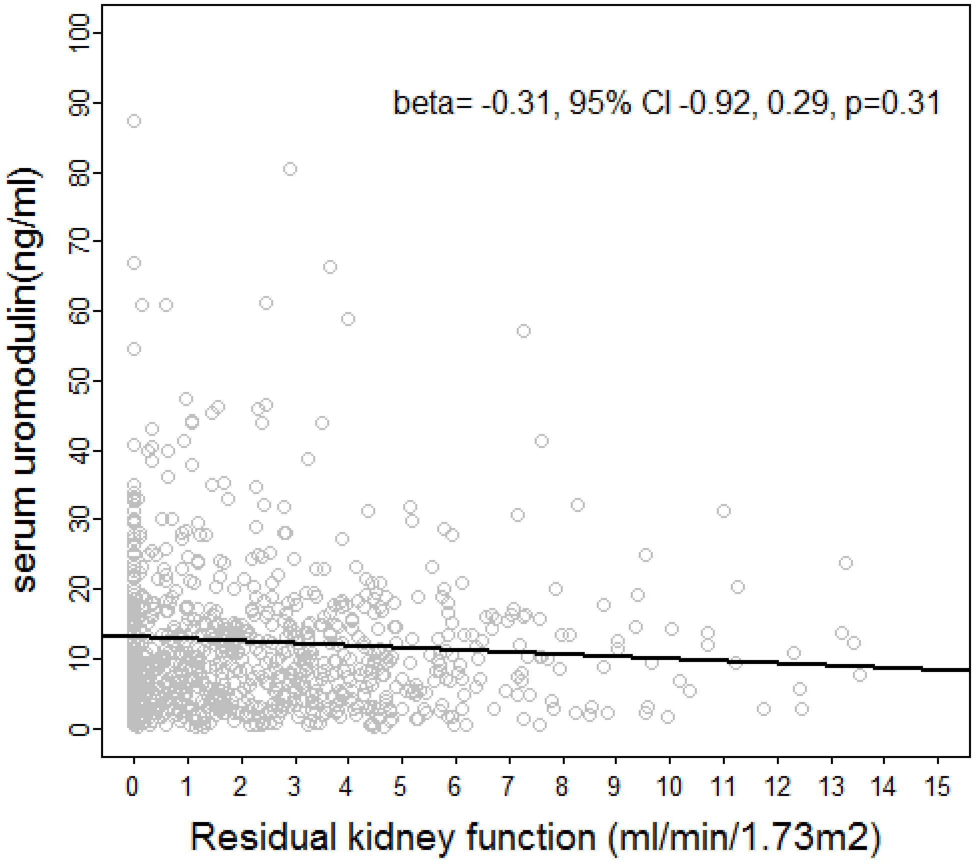

Figure S2: Kaplan-Meier survival curves according to serum uromodulin quartiles

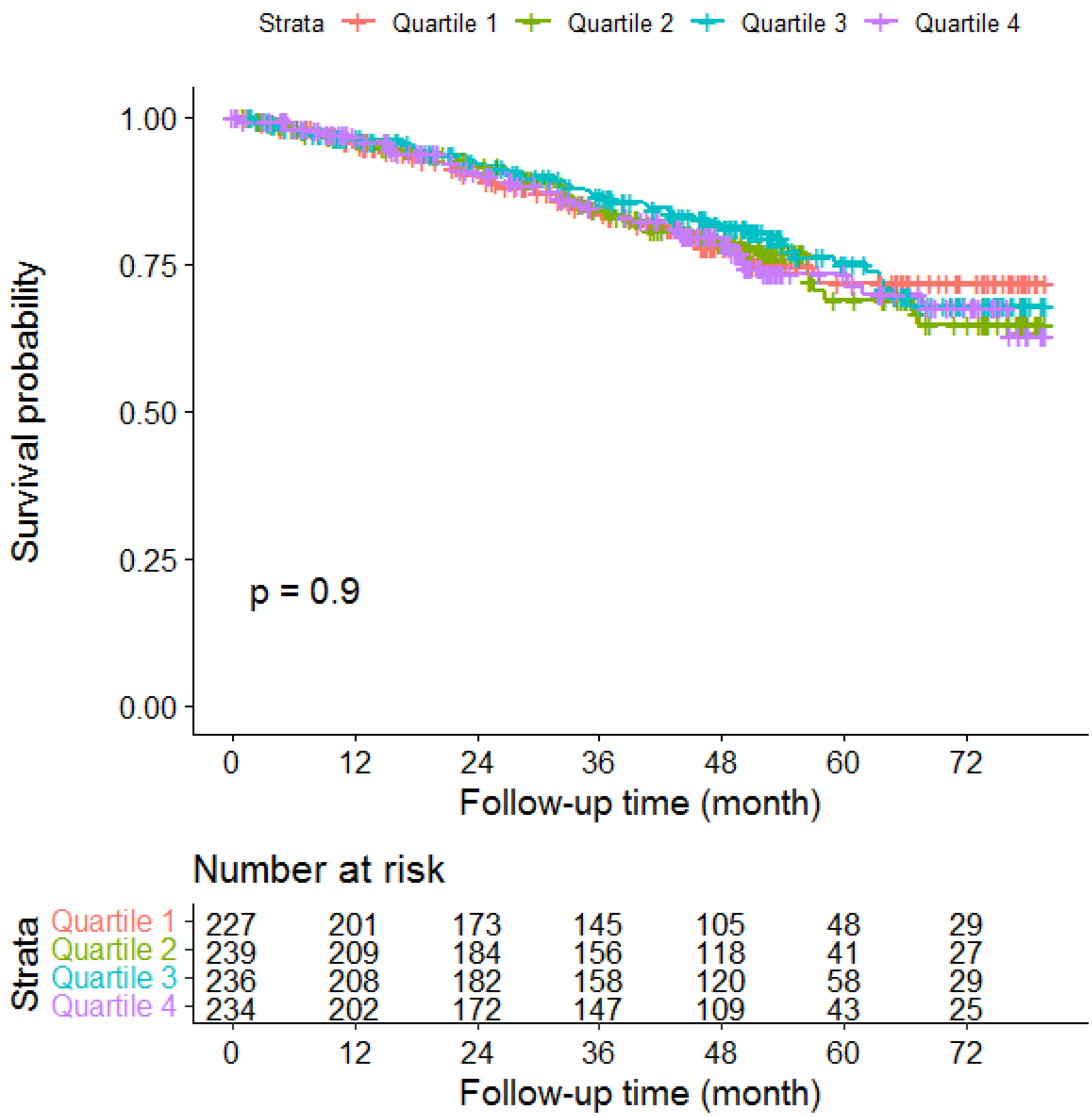

Item S1. Patient selection and definition, sampling procedure and of baseline and follow-up period

The Guangzhou PD Study consists of patients treated by continuous ambulatory peritoneal dialysis (CAPD) from the First Affiliated Hospital of Sun Yat-Sen University in Guangzhou and six tertiary care hospitals in southern China who were recruited between January 2013 and December 2015. Inclusion criteria were age ≥ 18 years and treatment with CAPD for ≥ 3 months. We also included anuric patients (i.e. residual urinary output <100 ml/day). Exclusion criteria were critical illness or major surgery at the time of study enrollment, active bleeding within the previous 3 days prior to enrollment, advanced stage of malignancy, peritonitis within 4 weeks prior to enrollment, untreated clinical disorders of the thyroid gland, and medications that block tubular secretion of creatinine. We further excluded patients with missing demographic variables and essential clinical data as well as missing samples on 24-h urine and/or dialysate collections. All serum samples were collected at the day of onsite follow-up visit and aliquoted within 24 hours of sample collection. Then samples were stored in refrigerator with -80 ℃ until uromodulin measurements occurred. Serum samples underwent one more freeze-thaw cycle before uromodulin measurements were performed. Baseline was defined as the individual time point of specimen collection for biomarker assessment. Serum samples were collected at the morning of the baseline visit for biomarker assessment when patients visited the outpatient clinic; PD was performed at home according to the routine PD regimen. Follow-up period was defined as the time from biomarker assessment to end of follow-up, which occurred either in case of death of end of data collection(August 31, 2019).

In statistical analyses, we assessed the correlation of sUMOD with residual kidney function (defined as the average mean of creatinine and urea clearance during a 24-hour urine collection) using Pearson correlation coefficient. Multivariable Cox proportional hazard regression analysis was applied to evaluate the association of sUMOD (both on a continuous scale and categorized into quartiles) with all-cause mortality. Due to skewed distribution, sUMOD was transformed into log scale when entered into the model as a continuous variable.

Table S1: Baseline characteristics overall and according to serum uromodulin quartiles

	**Overall (n=936)**	Quartile 1 (n=227)	Quartile 2 (n=239)	Quartile 3 (n=236)	Quartile 4 (n=234)	p for linear trend
Uromodulin (ng/ml)	9.2	2.4	6.5	11.7	21.1	
Median (25th, 75th)	(4.5, 15.2)	(1.5, 3.3)	(5.6, 7.9)	(10.3, 13.5)	(17.4, 28.6)	
Range (min, max)	(0.1, 777.8)	(0.1, 4.4)	(4.5, 9.1)	(9.2, 15.2)	(15.3, 777.8)	
**Demographics and baseline characteristics**						
Age (years)	49.7 ± 14.6	48.7 ± 14.5	50.1 ± 14.1	50.7 ± 15.5	49.3 ± 14.3	0.60
Female (n, %)	446 [47.6]	103 [45.4]	118 [49.4]	106 [44.9]	119 [50.9]	0.49
Cause of kidney failure (n, %)						0.44
Glomerular disease	*627 [67.0]*	*158 [69.9]*	*162 [67.8]*	*152 [64.4]*	*155 [66.2]*	
Diabetes mellitus	*148 [15.8]*	*38 [16.7]*	*40 [16.7]*	*36 [15.3]*	*34 [14.5]*	
Arterial hypertension	*78 [8.3]*	*18 [7.9]*	*20 [8.4]*	*19 [8.1]*	*21 [9.0]*	
Obstructive nephropathy	*51 [5.4]*	*9 [4.0]*	*12 [5.0]*	*19 [8.1]*	*11 [4.7]*	
Others	*32 [3.4]*	*4 [1.8]*	*5 [2.1]*	*10 [4.2]*	*13 [5.6]*	
BMI (Kg/m^2)	22.2 ± 3.2	22.2 ± 3.0	22.1 ± 3.2	22.2 ± 3.2	22.2 ± 3.5	0.80
SBP (mmHg)	140 ± 19	138 ± 20	141 ± 19	141 ± 19	140 ± 19	0.38
DBP (mmHg)	85 ± 14	84 ± 13	85 ± 13	85 ± 14	85 ± 14	0.47
**RKF & Dialysis-related variables**						
Dialysis vintage (months)	15.8 [2.2, 35.5]	15.3 [2.6, 32.6]	17.3 [3.0, 40.2]	14.9 [1.8, 34.4]	17.7 [2.6, 36.2]	0.97
Dialysate inflow (L/d)	7.7 ± 1.2	7.8 ± 1.0	7.8 ± 1.3	7.6 ± 1.3	7.7 ± 1.1	0.12
Dialysate outflow (L/d)	8.2 ± 1.4	8.3 ± 1.3	8.3 ± 1.6	8.0 ± 1.5	8.2 ± 1.3	0.23
Ultrafiltration (mL/d)	500 [150, 800]	500 [125, 805]	550 [200, 800]	445 [100, 800]	518 [175, 800]	0.86
Urinary output (mL/d)	500 [100, 900]	500 [100, 800]	500 [50, 800]	500 [208, 1000]	475 [50, 900]	0.39
RKF (ml/min/1.73m^2)	1.6 [0.3, 3.6]	1.5 [0.3, 3.4]	1.4 [0.2, 3.5]	1.9 [0.7, 3.8]	1.2 [0.1, 3.5]	0.43
PD clearance (ml/min/1.73m^2)	4.7 [4.0, 5.1]	4.6 [4.0, 5.1]	4.7 [4.1, 5.2]	4.6 [3.9, 5.0]	4.7 [4.1, 5.1]	0.52
Total clearance (ml/min/1.73m^2)	6.5 [5.3, 8.1]	6.4 [5.3, 7.9]	6.4 [5.2, 8.0]	6.7 [5.5, 8.5]	6.3 [5.2, 8.0]	0.28
**Laboratory values**						
Creatinine (mg/dl)	10.3 ± 3.5	10.4 ± 3.5	10.3 ± 3.3	9.9 ± 3.2	10.3 ± 3.8	<0.001
Phosphorus (mmol/L)	1.5 ± 0.5	1.6 ± 0.5	1.6 ± 0.5	1.5 ± 0.4	1.6 ± 0.6	0.99
Potassium (mmol/L)	4.0 ± 0.7	4.0 ± 0.7	4.0 ± 0.7	3.9 ± 0.7	4.0 ± 0.7	0.65
Albumin (g/dL)	3.7 ± 0.5	3.7 ± 0.5	3.7 ± 0.5	3.7 ± 0.5	3.7 ± 0.4	0.13
Total cholesterol (mmol/L)	5.0 ± 1.2	5.0 ± 1.1	5.1 ± 1.3	5.1 ± 1.3	4.9 ± 1.2	0.38

C-reactive protein (mg/L)	1.9 [0.7, 5.1]	2.0 [0.6, 4.8]	1.9 [0.6, 4.4]	2.0 [0.8, 5.8]	2.0 [0.6, 5.1]	0.75

Values in mean ± standard deviation or median [25%-, 75%-interquartile range], respectively. Abbreviations: BMI = body-mass-index; DBP = diastolic blood pressure; PD clearance = peritoneal dialysis clearance, defined as the average mean clearance of urea and creatinine; SBP = systolic blood pressure; RKF = residual kidney function, defined as the average mean clearance of urea and creatinine; Total clearance defined as the sum of RKF and PD clearance, defined as the average mean of urea and creatinine clearance

Table S2: All-cause mortality event rates according to serum uromodulin quartiles in the PD cohort vs. previously published events rates in patients with chronic kidney disease, categorize into quartiles according to serum uromodulin concentrations

	Chinese PD cohort					GCKD cohort (4)				
	Total cohort (n=936)	Q1 (n=227)	Q2 (n=239)	Q3 (n=236)	Q4 (n=234)	Total cohort (n=5143)	Q1 (n=1286)	Q2 (n=1287)	Q3 (n=1285)	Q4 (n=1285[illegible]
sUMOD range (in ng/ml, min-max)	0.1-777.8	<4.5	>=4.5 - <9.1	>=9.2 - <15.225	>=15.225	0.0-490.4	<=55.6	>55.6-83.4	>83.4-125.3	>125.3[illegible]
Events (n, %)	195 (20.8)	47 (20.7)	51 (21.3)	47 (20.0)	50 (21.4)	335 (6.5)	145 (11.3)	86 (6.7)	63 (4.9)	41 (3.2[illegible]
Follow-up time (months)	46.8 [25.9, 54.0]	46.1 [25.6, 53.9]	47.8 [28.2, 54.0]	48.5 [26.8, 59.8]	45.9 [22.6, 53.0]	n/a	n/a	n/a	n/a	n/a
Incidence per patient year, (%)	5.9 [5.1, 6.7]	5.8 [4.3, 7.6]	6.0 [4.5, 7.8]	5.4 [4.0, 7.1]	6.2 [4.3, 7.7]	1.1 [0.8, 1.4]	1.7 [1.2, 2.3]	0.9 [0.6, 1.4]	1.0 [0.7, 1.4]	0.6 [0.4, 0.9[illegible]

Follow-up time presented as median (25th, 75th); PD, peritoneal dialysis; sUMOD, serum uromodulin

Table S3 Cox regression analysis to evaluate the association of serum uromodulin with cardiovascular and non-cardiovascular mortality in peritoneal dialysis patients (n=936)

	Events (%)	Univariable HR (95% CI)	Model 1* HR (95% CI)	Model 2† HR (95% CI)	Model 3‡ HR (95% CI)
Cardiovascular death					
Increase per one unit of log serum uromodulin higher	100 (10.7)	1.10 [0.90, 1.34]	1.06 [0.86, 1.31]	1.05 [0.85, 1.29]	1.04 [0.84, 1.29]
Q1	21 (9.3)	Reference	Reference	Reference	Reference
Q2	32 (13.4)	1.45 [0.83, 2.51]	1.28 [0.73, 2.22]	1.28 [0.73, 2.23]	1.28 [0.73, 2.23]
Q3	24 (10.2)	1.05 [0.59, 1.89]	0.86 [0.48, 1.57]	0.87 [0.48, 1.57]	0.87 [0.48, 1.57]
Q4	23 (9.8)	1.10 [0.61, 1.99]	1.08 [0.60, 1.96]	1.03 [0.57, 1.88]	1.03 [0.57, 1.88]
Non-cardiovascular death					
Increase per one unit of log serum uromodulin higher	95 (10.1)	0.97 [0.80, 1.18]	0.89 [0.73, 1.11]	0.89 [0.72, 1.09]	0.89 [0.72, 1.10]
Q1	26 (11.5)	Reference	Reference	Reference	Reference
Q2	19 (7.9)	0.69 [0.38, 1.25]	0.63 [0.35, 1.16]	0.63 [0.34, 1.16]	0.63 [0.34, 1.15]
Q3	23 (9.7)	0.81 [0.46, 1.43]	0.62 [0.35, 1.11]	0.63 [0.35, 1.13]	0.61 [0.34, 1.09]
Q4	27 (11.5)	1.05 [0.61, 1.80]	1.00 [0.58, 1.73]	1.00 [0.57, 1.73]	0.99 [0.57, 1.72]

HR: hazard ratios.

Non-cardiovascular death included death due to infectious disease (n=37), other causes (n=29) and unknown cause (n=29).

Serum uromodulin was evaluated on a logarithmic scale as a continuous variable and on raw scale for categorization into quartiles.

Serum uromodulin Quartile distribution: Quartile 1 (Q1)< 4.5 ng/ml, Quartile 2 (Q2)≥ 4.5 and <9.2 ng/ml, Quartile 3 (Q3) ≥ 9.2 and <15.225 ng/ml, Quartile 4 (Q4) ≥ 15.225 ng/ml.

* adjusted for age, sex, body-mass-index, diabetes, systolic blood pressure, serum phosphorus, serum potassium, serum albumin, serum c-reactive protein, serum total cholesterol

† Model 1 + peritoneal ultrafiltration, peritoneal average mean of urea and creatinine clearance, renal average mean of urea and creatinine clearance

‡ Model 2 + dialysis vintage
